# Notes and News

**Published:** 1900-06-02

**Authors:** 


### Notes and News.

A fatal case of bubonic plague was reported at Durban last week. The victim was an Indian.

The University of California is fortunate in its patroness, Miss Phoebe Hearst, who has presented it with a complete equipment for the department of pathology.

Princess Henry of Battenberg has consented to lay the foundation-stone of the new Belgrave Hospital for Children in Clapham Road, Kennington, on Wednesday, June 27th.

On Saturday last the Duke of Cambridge laid the memorial-stone of a new wing of the Victoria Hospital at Folkestone, the addition being a memorial of the sixtieth year of the Queen's reign. The new wing will cost about £6,000, of which all but about £900 has been subscribed.

In consequence of reports concerning the sanitary condition of the various European countries, the Italian Government recently suggested the advisability of a temporary suspension of the pilgrimages to Rome connected with the Holy Year, and the Pope has arranged that all such pilgrimages shall be suspended until September 15th.

The new Indian order for public service, the Kaiser-i-Hind medal, has been presented to Lieut.-Colonel R. N. Campbell, Civil Surgeon for Shillong, and Captain C. H. James, Plague Medical Officer in the Jullundar and Hoshiarpur District. These medals are gold and of the first-class. Captain J. W. Grant, of the Indian Medical Service, now on plague duty at Rajpootana, receives the silver medal, and so also does Miss C. Adams, who is a medical practitioner in India.

It is proposed to introduce the "closure" into the debates of the General Medical Council. Under the present standing orders a motion may be carried, or rejected, or modified, or it may be passed over by going to the next order of the day. But there is no power by which to close the debate, and at the same time to take a vote on the subject under discussion. If the proposal should be accepted there can be no doubt that much time will be saved, which, to use the President's words, is at present wasted "in the academic discussion of questions not ripe for a practical solution."

The crusade against alcoholism in France is growing; not only is alcohol prohibited in military canteens, but in civil circles an effort is to be made to secure the registration of deaths directly traceable to alcoholism under this head.

The new medical departure in Birmingham, under the auspices of the Hospital Saturday Fund, entitled the Consultative Medical and Surgical Institution, has commenced operations. A large advertising placard is supplied to the workshops, which bears the information that consultations can be secured for the fee of half-a-guinea at the institute.

In answer to Sir J. Leng, who asked last Monday what provision had been made for invalided soldiers from South Africa, who were so physically weak as to be incapacitated from earning a livelihood, Mr. Wyndham stated that the men will be paid and medically treated until they are discharged, their families meanwhile receiving the ordinary allowances. After their discharge their claims to pension will be considered by the Chelsea Commissioners. It is to be noted that the powers of the Commissioners have recently been enlarged to admit of a higher scale of pension being accorded to men incapacitated by disease contracted on service.

A select committee has been appointed by Government to consider the operation of the law by which hospitals and other institutions for the care and treatment of the sick or of those afflicted in mind or body are liable to local rates, and to report whether under any and what conditions it is for the public interest that such hospitals and institutions, or any of them, should be exempted wholly or in part from such liability in future. The committee consists of Mr. Bonsor, Dr. Farquharson, Mr. Hayes Fisher, Sir Cameron Gull, Sir John Maclure, Mr. M'Crae, Mr. Pickersgill, Mr. Round, Mr. T. W. Russell, Mr. Tomlinson, Mr. Lawson Walton, Mr. Warr, and Sir James Woodhouse.

On going into Committee of Supply last week, Mr. Dillon referred to the action of the Local Government Board of Ireland, in the case of the Ballymena Guardians. The Board had, he said, issued a ukase to the guardians directing them to allow their medical officer a month's holiday, and to pay a substitute, but the Ballymena Board appealed against the order, and the Chief Baron had, he said, decided that it should be quashed, as the Local Government Board had exceeded their powers in the matter. In answering him, however, Mr. Gerald Balfour, while admitting that a mistake had been made, and that the Local Government Board had not drafted their rule in such a form as to give them the power they desired, held that the judges had not laid it down that the Board had no power to enact such a rule, or that such a rule would not be a proper one. The best way, he said, to cure any mistake that might have been made would be to alter the rule, and that no doubt would be done in due course. This puts the matter in a proper light. It was a scandal that medical officers should by the action of the local authorities be deprived of holidays promised them under the sanction and guarantee of the central authority.

